# To examine environmental pollution by economic growth and their impact in an environmental Kuznets curve (EKC) among developed and developing countries

**DOI:** 10.1371/journal.pone.0209532

**Published:** 2019-03-26

**Authors:** YuSheng Kong, Rabnawaz Khan

**Affiliations:** School of Finance and Economics, Jiangsu University, Zhenjiang, Jiangsu, People’s Republic of China; The Bucharest University of Economic Studies, ROMANIA

## Abstract

This study analyzes the core energy consumption among countries’ specific variables by Environmental Kuznets Curve hypothesis (EKC), for a panel data of 29 (14 developed and 15 developing) countries during the period of 1977–2014. By assessing Generalized Method of Moments (GMM) regressions with first generation tests such as common root, individual Augmented Dickey-Fuller (ADF), and individual root-Fisher-PP which have been computed individually, the results confirm the EKC hypothesis in the case of emissions of solid, liquid, gases, manufacturing industries and also construction. Hence, we computed the cointegration test by Pedroni Kao from Engle-Granger based and Fisher. Since the variables are co-integrated, a panel vector error correction model is estimated in GDP per capita, emission from manufacturing industries, arms import, commercial service export, and coal rent, in order to perform Pairwise Granger Causality test and indicate Vector Error Correction (VEC), with co-integration restrictions. Moreover, the statistical finding from VEC short-run unidirectional causality from GDP per capita growth to manufacturing industries and coal rent, as well as the causal link with manufacturing industries and commercial service export. Additionally, there occurred no causal link among economic growth, arm import and coal rent.

## 1. Introduction

**Fig 1:**
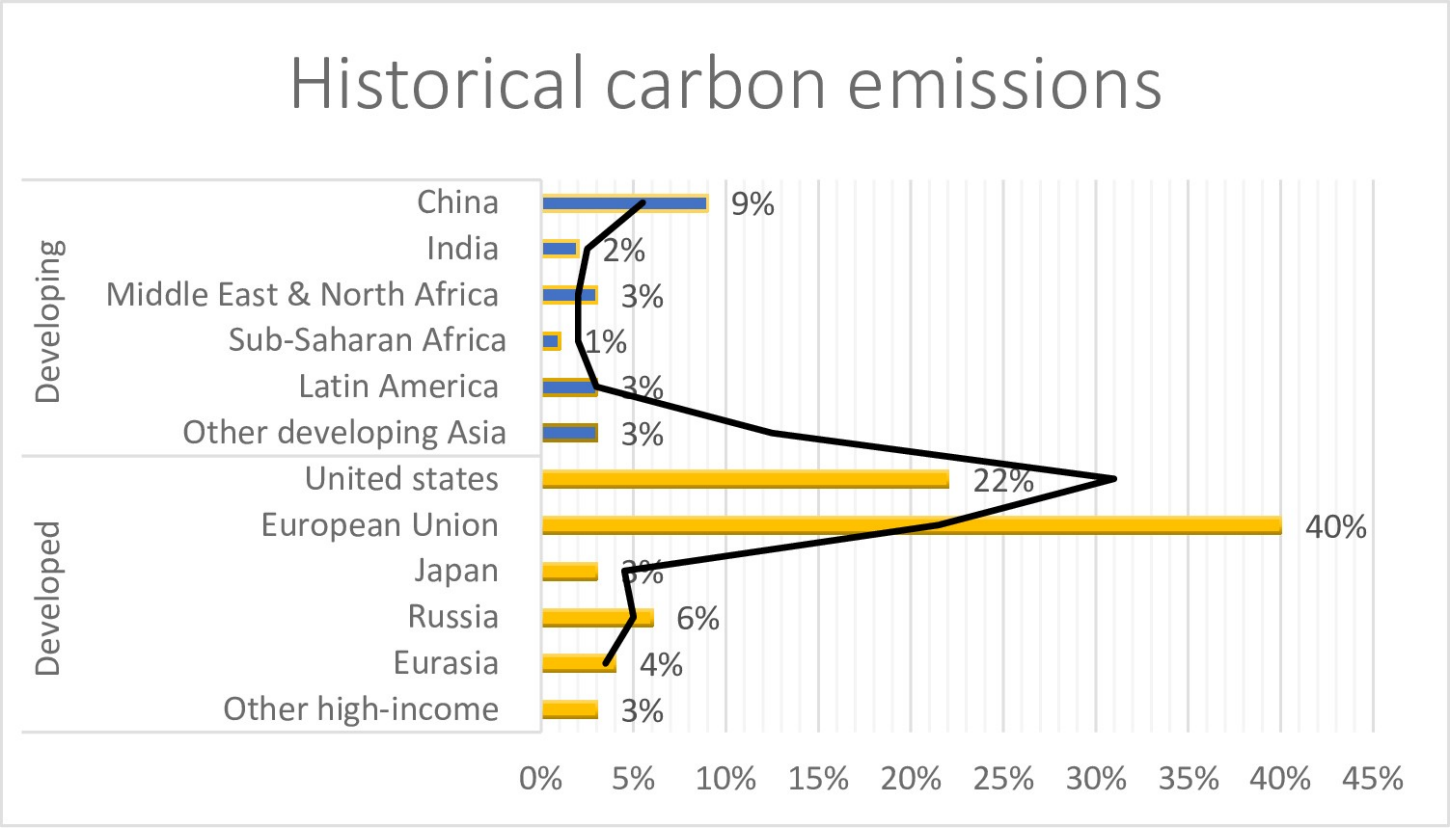
Historical carbon emission. Source: LUCEF, 1850-2011(CAIT v2.0).

Developing countries, with the rapid development of economy, are leading the growth of energy consumption globally. The energy consumption of developing countries was 7.64×10^9^ (ton) oil equivalent (toe), accounting for 58.1% in 2005 all over the world, also in 2015 the consumption of energy increase in developing countries by 2.38×109 (toe). The level of energy intensity in China (8.34), Russia (9.49) and Germany (3.88), indicate a big gap between developing and developed countries. Another side the developing countries decrease energy intensity slowly and try to achieve the bottleneck problems with well-developed technology [1,2]. Furthermore, 79% of developed countries are responsible for historical carbon emission, in which the USA is 22%, the European Union is 40% and China is 9% Fig 1. There are 60% of CO2 emission responsible countries are China and USA, it’s is two-fifth and these top polluters do about the heat-trapping gases liable for global warming and their infections. Also in 2013 CO2 emission is 11 billion tons with 1.36 billion population. The 62% coal consumption cap has been announced by 2020 in China. The China and USA deal on greenhouse gas emission growth by 2030, while its significant and also little effected on the global thermostat. The USA government estimates China doubling it emission by 2040 cause of major changes and reliant on fossil fuels for steel and electricity production. There was 2.6 billion tons CO2 emission in India with 1.2 billion population, 2 billion tons in Russia with 143.5 million population, 1.4 billion tons in Japan with 127 million population, 836 million tons in Germany with 80.6 million population in 2013.

The solid fuel consumption varies in different countries regarding with magnitude of indicators, the darker shade, and higher the value. The China highest value in all over the world is 7,431,146.00. Bolivia is the lowest value with 0.00. CO2 is naturally occurred with gas fixed by photosynthesis into organic matter, also biomass burning and the byproduct of fuel consumption of fossil emitted from land use to changes along with industrial processes. The industrial revolution has rapidly increased global warming and atmospheric carbon dioxide [3]. Burning wood, oil, coal and waste material, such as in the industrial process of cement has been increased CO2 emission.

The USA is one of a top developed country by CO2 emission from gaseous fuel consumption in all over the world and 1.43 million kt that account for 21.72% of world’s CO2 emission from gaseous fuel consumption in 2014. Other five top countries (China, Russian Federation, Iran, and Japan), 48.97% account of it. In 2014, estimated emission of CO2. from fuel gaseous was at 6.6 million. Furthermore, it’s injected into the melting zone, auto-ignited (Solid combustion zone) and the methane concentrations of 0 to 5% vol, also the total calorific heat input unchanged. The pattern of heat in the melting zone was recorded by non-contact thermal infrared imager and thermocouples. Significantly, the result indicated that extend the melting zone from the upstream and it higher than from coke sintering, without increasing the energy consumption. Therefore, the saving potential was evaluated by reducing the heat 4 to 8%. [4,5]. The continue modification and well-developed technology have been directly affected by solid combustion zone, like 15% energy consumption in the iron and steel industry in China and 26% consumption in the pre-treatment process. The CH4 emission was approximately 5.1million tones, equivalent to 10.78 million of CO2, it indicated the third largest source of CH4 emission.

Municipal solid waste (MSW) landfills 69% of the solid waste which received from USA (94% of total landfills emission). Furthermore, the waste of energy emission was accounted 12.1 million metric tonnes of CO2 emission competitively 1745 million emitted in the field of transportation.

**Fig 2:**
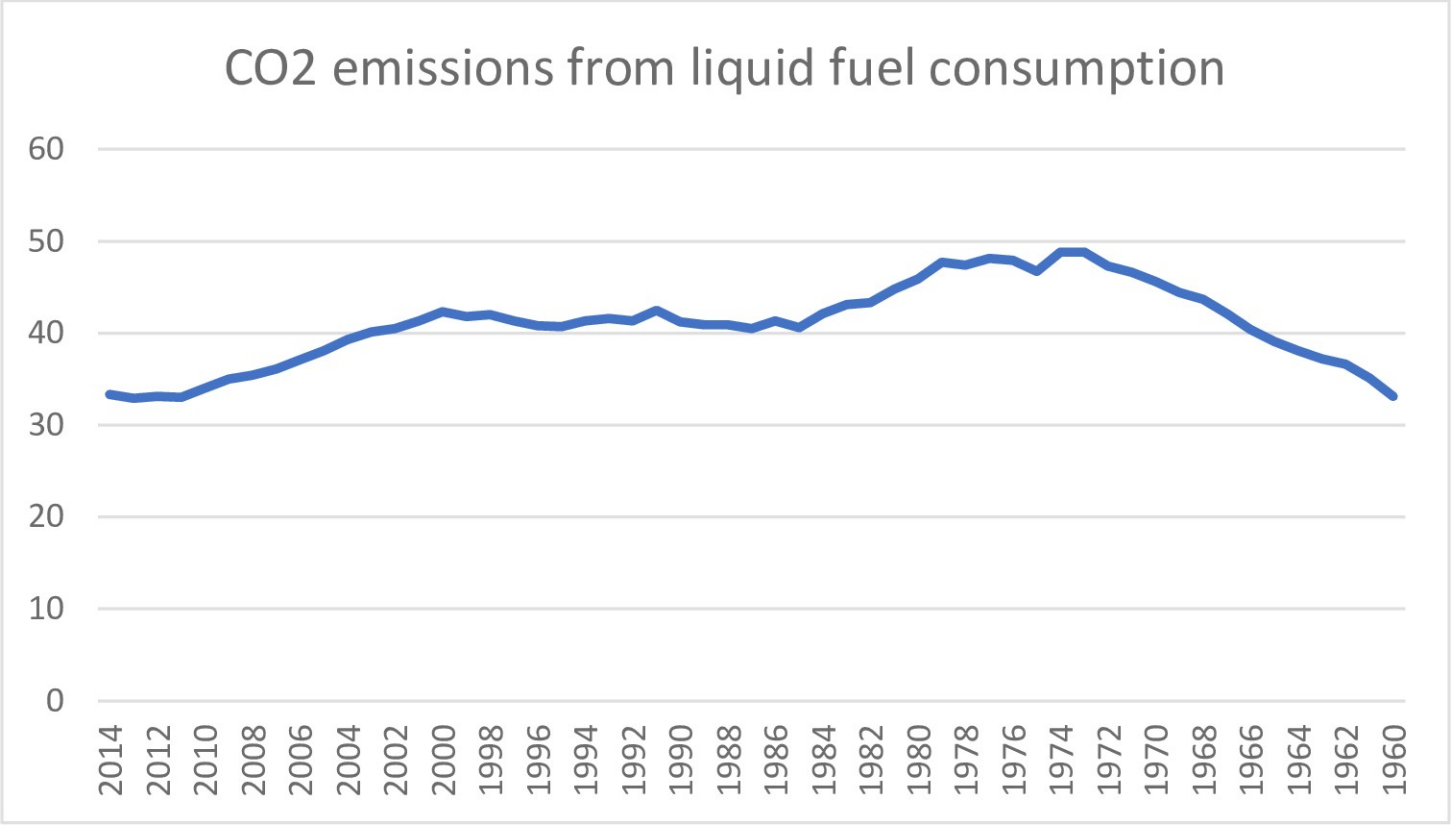
CO2 emission from liquid fuel consumption. Source: Authors’ amplification.

While 26.5 million tonnes incineration is used to treat waste in the USA, or approx. 7 to 19 percent of solid waste generated. Meanwhile, 3.2% CO2. emission have been increased in 2010 and total greenhouse gases were equivalent to 6.82% billion metric tonnes of CO2. While CO2 is found in our environment but the problem is that the industrial revolution has increased the quantity of it in the 19^th^ century by industrial modification Fig 2, because it’s most prominent greenhouse gases climate change and most of the scientists agree on that is not only for Chinese hoax. The Carbon dioxide information analysis center (CDIAC), realized more than 400 billion metric tonnes in atmosphere from fossil consumption and especially production of cements since 1751. Also, the combustion of solid and liquid fossil fuel causes of 4^th^ of all CO2 which is 9.9 billion tones in 2014.

Environmental Kuznets Curve (EKC) has been already explored different ideas in CO2 emission. The EKC growth strategy is to grow now and clean later is the too much intensive resource and huge environmental cost and developing countries should follow the growth path than that of EKC.[6–8] In the emerging economies a substantial fraction of the production satisfies the consumption in developed countries cause of the notorious carbon leakage problem and embodied of carbon emission in exports not contacted in the production-based emission accounting (PBA) [9]. The U-shaped of EKC is the relationship between income and environmental degradation and it increases as income increases and declines after income exceeds, suggest that growth is the cause and cure of air pollution [10]. However, the consequence of economic growth and trade policies should the align with energy sector [11]. The economic growth is not suitable and for environmental protection so, therefore, we should lower the economic growth. [12–14].

Such EKC tested for historical perspective along with fuel prices and growth in Sweden in the period of 1870–1997[15]. Explored the energy consumption and study of the electricity in Saudi Arabia with Time-Varying parameters vector autoregressive (TVP-VAR) in the period of 1970–2010. [16]. Study the dynamic impact and economic output and Carbon emission from 1991–2012[17]. Tested the EKC hypothesis for the solid waste generation with panel data from 1997–2010 in 32 European states [18]. Studied the technological progress and EKC, associated with economic growth and CO2 emission in panel data in 24 European nations from 1990–2013 [19]. Explored the transport energy by using EKC with the hypothesis in EU-27 countries from 1995–2009 [20].

The main feature of this paper is to distinguish from others on the bases on research samples, as well as several part of emission apart from CO2, namely CEMIC (CO2 emission from manufacturing industries and construction), AET (Arms export trend indicator), AIT (Arms import trend indicator), CSE (Commercial service export), IF (Insurance and financial service), CR (Coal rents (GDP)) and MIE (Military expenditure). Also, this study is unique on the bases of economic growth, Likewise, in decoupling of economic growth and CO2 emission in developing and developed on seven state, industrialization of CO2, renewable and non-renewable energy of 42 developing economy, three groups of renewable energy and southwest economic zone CO2 emission in China [21–25] has not indicated the 29 (14 developed and 15 developing) countries. Furthermore, how developing countries are creating effects on CO2 emission on other developed countries and how the manufacturing industries and military expenditure effects on the CO2 emission. The following logical structure and literature highlighted the EKC hypothesis along with the relationship between CO2 emission and economic growth. Section 3 is presented the data analysis with the econometric framework. Section 4 are shown empirical result and discussion, while the final section of the paper concludes and provides implication policies with recommendations.

## 2. Literature review

Catholic part of specific literature explores the association between EKC and the national income of the countries, and greater environmental quality and their effects on developed and developing countries Table 1. According to Kuznets’ inverted U-hypothesis, initial stage as per capita national income of countries rise, inequality in income distribution rises after reaching the highest degree, where the country develops and it is per capita income automatically rises in maximum level, and it falls as GDP per capita increases further [26]. Explored the study of 1955, and calculated the Kuznets’ ratio and found that, whereas developed countries tend to have a lower degree of inequality, the developing countries tend to have a higher degree of inequality [27]. That the evidence of inverted U-hypothesis, regarding the relationship between economic growth and inequality. It means those income inequalities where higher in developing countries compared to developed countries, but after that in particular stage, increase in economic growth will reduce the environmental pressure.

**Table 1:** Literature review of economic growth and CO2 emission.

Study	Datasets	Econometric techniques	Period	Outcomes
[28]	12 Western European countries	linear cointegration model	1861–2015	Elasticity of income of CO2 emission in all countries. The cointegration method of CO2 emission and GDP of countries. The study important for developing countries.
[29]	Tunisian	Vector Autoregressive (VAR) model.	1980–2014	Determined the influence factor of CO2 emission. Explored, the EKC with inverted U-shaped pattern in CO2 emission.
[30]	21 industrial countries	Unit root test	1960–1997	The test result was consistent with narrow and wide application in different industrial countries.
[31]	21 OECD countries	Univariate unit root tests	1950–2014	The per capita CO2 emission is less explosive at each quantile without smooth break in 21 OECD Countries.
[32]	Pakistan	ARDL approach	2014	Dynamic causality between energy consumption, economic growth and CO2 emission.
[33]	South African	ARDL approach, Engel Granger method.	1960–2009	Per capita has significant long positively effect in level of CO2. Find bidirectional causality between in income per capita and foreign trade.
[34]	116 Countries	Panel vector autoregressive (PVAR), Generalized method of moment (GMM)	1990–2014	Energy consumption does not cause of regional level, Economic growth has negative casual impact on carbon emission, energy consumption positively causes of economic growth in sub-Saharan Africa.
[35]	28 subsectors	Generalized Method of Moments (GMM)	2002–2015	FDI is positive predictor of environmental quality and reduce CO2 emission level.
[23]	42 developing countries	Granger causality modeling, error correction model (ECM), Generalized Method of Moments (GMM)	2002–2011	In long the energy consumption positively contribute to economic growth.
[36]	India, Indonesia, China and Brazil	Autoregressive Distributed Lag (ARDL)	1970–2012	EKC finding that Brazil, China and Indonesia impact on income and reduce their CO2 emission.
[37]	24 sub-Saharan African countries	Panel cointegration	1980–2010	Inverted U-Shaped EKC is not supported for these countries in long-run estimation; export have a positive and import have a negative impact on CO2 emission.
[38]	China and India	ARDL	1965–2013	EKC result supported by long-run positive impact on emission
	20 countries in Middle East and North Africa (MENA)	Regression	1980–2014	EKC impact by regression on population, affluence and technology framework.
[19]	5 economies of South Asia	FMOLS	1971–2013	Consumption of energy and population density will increase in long run.
[39]	14 Asian countries	GMM	1990–2011	To support EKC by emissions and income per capita and results are statistically significant.
	Middle East, North Africa, Sub-Saharan Africa	DOLS and VEC	1980–2010	The results of EKC indicate significance of renewable energy consumption.
[40]	25 OECD countries	FMOLS	1980–2010	EKC verified that non-renewable energy CO2 emissions renewable.

Sources: Authors’ compiling by the literature review

The EKC point starting from [41] showed that there is an inverted U-Shaped and relationship between per capita income and energy intensity in 173 countries and found CO2 emission by error correction model [42]. Explored the EKC hypothesis for a panel of 20 countries with traditional inverted U-shaped relationship. [43] That study empirically related to economic and population growth and CO2 emission from 1990 to 2014. The cross-sectional study results dependent on slop homogeneity and heterogeneity. The common correlated effect means a group (CCEMG), indicated the population size, economic growth and the significant influence on the level of CO2 emission.

## 3. Data and methodology

### 3.1 Sample and variables

The data sample covers the period of 1977–2014 for a panel consisting of the 29 (14 developed and 15 developing) countries. Table 2 indicate the variables, used for analysis, as well as their definitions and the sources of data, are presented with different abbreviations. A part of preceding studies the EKC have already treated with different variables, like consumption of energy and economic growth. [23,28,32,42,44], while the other new variables such as corruption, electricity consumption, population urbanization, industrial revolution provides more consideration [30,45]. In CESFC, CEGFC, CELFC, CE, CEMIC control the trend of explanatory variables of AIT, CSE, IGD, CR, IF, ME and AL as well, high technology manufacturing sector includes high skill labor contribution in development and creating the significant effects on the economy. Furthermore, Table 3 summarized the turning points to identify the earlier studies.

**Table 2:** Variables description for the analysis.

Variables	Definition	Unit measurement	Time frame availability	Data sources
GDP	GDP per capita	Constant 2010 US dollars	1977–2017	World Bank (NY.GDP.MKTP. KD)
GDPC	GDP Per capita growth	Annual %	1977–2017	World Bank (NY.GDP.PCAP.KD. ZG)
CESFC	Co emissions from solid fuel consumption	kt	1977–2014	World Bank (EN.ATM.CO2E.SF.KT)
CEGFC	Co emissions from gaseous fuel consumption	kt	1977–2014	World Bank (EN.ATM.CO2E.GF.KT)
CELFC	Co emissions from liquid fuel consumption	kt	1977–2014	World Bank (EN.ATM.CO2E.LF.KT)
CE	Co emissions	kt	1977–2014	World Bank (EN.ATM.CO2E. KT)
CEMIC	Co emissions from manufacturing industries and construction	% of total fuel combustion	1977–2014	World Bank (EN.CO2.MANF. ZS)
ME	Merchandise Export	% of total merchandise exports	1977–2016	World Bank (TX.VAL.MRCH. R2. ZS)
AET	Arms export trend indicator	Value	1977–2017	World Bank (MS.MIL.XPRT. KD)
MI	Merchandise Import	% of total merchandise imports	1977–2016	World Bank (TM.VAL.MRCH. R2. ZS)
AIT	Arms import trend indicator	Value	1977–2017	World Bank (MS.MIL.MPRT. KD)
CSE	Commercial service export	Current US dollar	1977–2017	World Bank (TX.VAL.SERV.CD.WT)
IGD	inflation GDP deflator	Annual %	1977–2017	World Bank (NY.GDP.DEFL.KD. ZG)
CR	Coal rents (GDP)	% of GDP	1977–2016	World Bank (NY.GDP.COAL. RT. ZS)
IF	Insurance and financial service	% of commercial service exports	1977–2017	World Bank (TX.VAL.INSF.ZS. WT)
MIE	Military expenditure	% of GDP	1977–2017	World Bank (MS.MIL.XPND.GD.ZS)
AL	Agriculture land	% of land area	1977–2015	World Bank (AG.LND. AGRI. ZS)

Sources: Selection based on databases’ availability

**Table 3:** Turning points reached earlier studies by pollutant type.

Pollutant types	Study	Datasets	Period	Econometric techniques	Turning points
CO2 emission		173 countries	1990–2014	Error correction model	(402,125.361 US$)
CO2 emission		20 countries	1870–2014	Bivariate model	$18,955 and $89,540 (in 1990 US$)
CO2 emission		128 countries	1990–2014	cross-sectional dependence and slope homogeneity tests	Significant
CO2 emission		141 countries	1970–2014	Spatial Green Solow model	Statistically significant
CO2 emission		India	1970–2015	autoregressive distributed lag (ARDL)	USD 2937.77
Renewable energy		Pakistan	1970–2014	autoregressive distributed lag (ARDL)	Significant
CO2 emission		27 Chinese cities	2001–2005	Panel data parameter estimation	34,328 CNY and 47,669 CNY
Industrial CO2 emission		USA	1973–2015	multilevel mixed-effect	Significant
CO2 emission		China	1995–2011	Input-output analysis	Significant
Fuel energy consumption		East Asian and Pacific countries	1990–2014	Generalized Method of Moment (GMM)	$5112.65

Sources: Authors’ compiling by the literature review

While MI and AET control the GDP, high manufacturing and export development creating negative aspects. Initially per capita increase the wealth also increases the CO2 emission. However, arms import has created also significant effects on CESFC, CEGFC, and CEMIC but not creating effects on CE Table 4. In empirical methodology, in what we follow, we start by testing unit roots all explanatory variables individually in panel data. If the variables have found non-stationary, we investigate the prevailing long run cointegration relationship and investigate their magnitude for long-run stationary. We employ a class of panel unit root test and panel cointegration test individually on all explanatory variables, which allow the serial correlation among the cross-section, i.e. the so-called second-generation test. Augmented IPS used by cross sectional [46] panel unit root test by Pesaran (2007) and as for panel cointegration used error-correction by Westerlund (2007), which both account for possible cross-sectional dependencies for individual explanatory variables. The key variables- CO2 emission of GDP (Constant 2010 US dollars) and per capita GDP (Annual %) growth along with other explanatory variables—in for both level and first difference. In the level case, we are unable to reject the null hypothesis, except for the GDP per capita growth, CO2 emission, arm import trend, commercial service export, and inflation GDP deflator.

**Table 4:** GMM regression with AB in n-Step.

	Dependent variables				
IDV	CESFC (1)	CEGFC (2)	CELFC (3)	CE (4)	CEMIC (5)
GDP	13.417***	16.319***	2.557***	-0.429***	6.731***
GDPSQ	-7.539***	-1.868*	-1.266*	-0.535*	-4.481***
ME	-1.565*	0.238*	-0.468*	0.115*	-3.367***
AET	45.327***	15.195***	2.804***	0.446*	11.343***
MI	2.772***	-0.602*	-0.123*	-0.286*	0.017*
AIT	12.944***	2.188***	1.809**	-0.857*	3.257***
CSE	-5.080***	2.945***	-4.878***	-0.436*	-1.963***
IGD	-0.739*	0.368*	-0.776*	-0.532*	0.274*
CR	27.038***	-0.809*	-0.276*	1.053*	3.970***
IF	16.766***	-6.582***	2.311***	-0.833*	0.291*
MIE	-3.117***	-3.044***	-1.069*	-0.854*	-1.099*
AL	0.652*	0.465*	-0.756*	-0.429*	-1.755**
Sargan statistic	0.384	0.102	0.827	0.212	0.185
J-statistic	8.520	17.220	5.080	12.021	17.319
Obs	480	480	480	480	480
N Countries	29	29	29	29	29

Sources: Computation by authors. Note: Please see, Table 2 for the variable’s definition.

*** specifies the statistically significant at 1% levels.

** specifies the statistically significant at 5% levels.

* specifies the statistically significant at 10% levels.

### 3.2 Econometric methods

EKC hypothesis, we followed the approach of [23,34,41,42,47–50]. The long-run relationship between polluted emission, GDP per capita, merchandise export, arms export, merchandise import, commercial service export, inflation GDP, coal rent, insurance, and financial service, military expenditure and agriculture land, is given as follows:

$$PE_{it}=a_{it}+\delta_{1i}PE_{it-1}+\delta_{2i}GDPC_{it}+\delta_{3i}{\left(GDPC_{it}\right)}^{2}+\delta_{4i}ME_{it}+\delta_{5i}AET_{it}+\delta_{6i}MI_{it}+\delta_{7i}AIT_{it}+\delta_{8i}CSE_{it}+\delta_{9i}IGD_{it}+\delta_{10i}CR_{it}+\delta_{11i}IF_{it}+\delta_{12i}ME_{it}+\delta_{13i}AL_{it}+\varepsilon_{it}\ldots \ldots \ldots .$$ (1)

Where PE shows the polluted emission and i = 1,….,29 and t = 1977,….,2014 reveal the country and time, respectively whereas emission, which we take from solid, gases, and liquid fuel, CO2 emission and CO2 emission from manufacturing industries and construction. *a*_*it*_ indicates the country fixed effect. The *δ*_1*i*_−*δ*_13*i*_ are parameters of long-run elasticities, which are related to each explanatory variable of the panel ε_*it*_, indicate estimated residuals, characterized for long-run equilibrium. Since the inverted U-shaped EKC hypothesis, ε_2*t*_ is expected to be positive and ε_3*t*_ is expected to be negative, also the monitoring value representing the turning points which is computed by *τ* = exp[−*β*_1_/(2*β*_2_)] [42,47,49]. Additionally, the research aims to establish the causal link between manufacturing industries and construction, economic growth, arms export, commercial service export and coal rent (GDP). Additionally, the Generalized Method of Moments (GMM) yields a steady and efficient parameter estimate in a regression, the explanatory variables are not strictly exogenous, heteroscedasticity and autocorrelation within existing [51]. The GMM is more efficient and effectual with an additional assumption that is the first difference in explanatory variables, which in turn allows the inclusion of more instruments. The GMM applied on 29 countries over 1977–2014 in order to analyze the impact of different explanatory variables on CO2 emissions. [52]. Thus, according to [53–55] first generation test such as common root-Levin, Lin (LLC), Chu and Breitung, individual (lm), Pesaran, shin (IPS), Augmented Dickey-Fuller (ADF), and individual root-Fisher-PP, and Hadri have been computed individually from all explanatory variables. Afterward, we computed the cointegration test by Pedroni, Kao from Engle-Granger based and Fisher (combined Johansen).

$$GDPC_{it}=a_{it}+\delta_{1i}t+{ϒ}_{1i}CEMIC_{it}+{ϒ}_{2i}AIT+{ϒ}_{3i}CSE_{it}+{ϒ}_{4i}CR_{it}+\varepsilon_{it}\ldots \ldots \ldots .$$ (2)

Where i = 1,….,29 and t = 1977,….,2014 for each country in panel data. Besides, the parameters *a*_*i*_ and *δ*_*i*_ indicate the fixed effect and deterministic trend. It is computing by Engle-Granger, long term model, specified in Eq (2) is estimated in which one period lagged and residual as an error correction term.

The dynamic error correction model is represented below:

$$△GDPC_{it}=a_{1j}+\sum_{k=1}^{q}\beta_{13ik}\;\Delta GDPC_{it-k}+\sum_{k=1}^{q}\beta_{14ik}\;\Delta CEMIC_{it-k}+\sum_{k=1}^{q}\beta_{15ik}\;\Delta AIT_{it-k}+\sum_{k=1}^{q}\beta_{16ik}\;\Delta CSE_{it-k}+\sum_{k=1}^{q}\beta_{17ik}\;\Delta CR_{it-k}+\partial_{1i}\epsilon_{t-k}+{Ʋ}_{1i}\ldots .$$ 3

$$△CEMIC_{it}=a_{2j}+\sum_{k=1}^{q}\beta_{23ik}\;\Delta GDPC_{it-k}+\sum_{k=1}^{q}\beta_{24ik}\;\Delta CEMIC_{it-k}+\sum_{k=1}^{q}\beta_{25ik}\;\Delta AIT_{it-k}+\sum_{k=1}^{q}\beta_{26ik}\;\Delta CSE_{it-k}+\sum_{k=1}^{q}\beta_{27ik}\;\Delta CR_{it-k}+\partial_{2i}\epsilon_{t-k}+{Ʋ}_{2i}\ldots .$$ 4

$$△AIT_{it}=a_{3j}+\sum_{k=1}^{q}\beta_{33ik}\;\Delta GDPC_{it-k}+\sum_{k=1}^{q}\beta_{34ik}\;\Delta CEMIC_{it-k}+\sum_{k=1}^{q}\beta_{35ik}\;\Delta AIT_{it-k}+\sum_{k=1}^{q}\beta_{36ik}\;\Delta CSE_{it-k}+\sum_{k=1}^{q}\beta_{37ik}\;\Delta CR_{it-k}+\partial_{3i}\epsilon_{t-k}+{Ʋ}_{3i}\ldots .$$ 5

$$△CSE_{it}=a_{4j}+\sum_{k=1}^{q}\beta_{43ik}\;\Delta GDPC_{it-k}+\sum_{k=1}^{q}\beta_{44ik}\;\Delta CEMIC_{it-k}+\sum_{k=1}^{q}\beta_{45ik}\;\Delta AIT_{it-k}+\sum_{k=1}^{q}\beta_{46ik}\;\Delta CSE_{it-k}+\sum_{k=1}^{q}\beta_{47ik}\;\Delta CR_{it-k}+\partial_{4i}\epsilon_{t-k}+{Ʋ}_{4i}\ldots .$$ 6

$$△CR_{it}=a_{5j}+\sum_{k=1}^{q}\beta_{53ik}\;\Delta GDPC_{it-k}+\sum_{k=1}^{q}\beta_{54ik}\;\Delta CEMIC_{it-k}+\sum_{k=1}^{q}\beta_{55ik}\;\Delta AIT_{it-k}+\sum_{k=1}^{q}\beta_{56ik}\;\Delta CSE_{it-k}+\sum_{k=1}^{q}\beta_{57ik}\;\Delta CR_{it-k}+\partial_{1i}\epsilon_{t-k}+{Ʋ}_{1i}\ldots .$$ 7

Where the first-difference operator indicates by Δ, the lag of length specified by *q* at one according to likelihood ratio test, and Ʋ specify serial uncorrelated error term.

## 4. Results

### 4.1 Descriptive statistics, correlation and unit root examination

**Fig 3:**
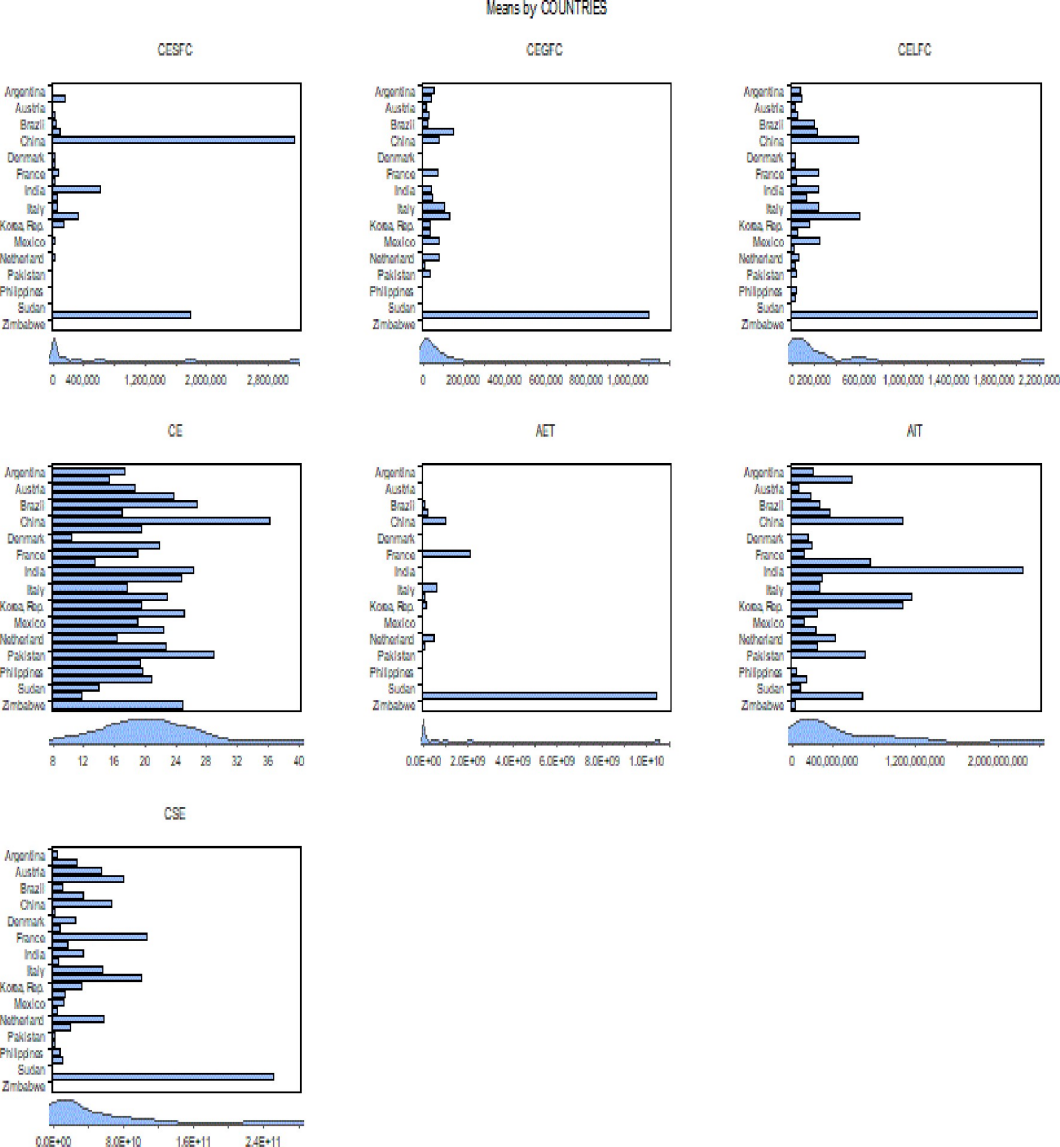
Highest mean valuation of pollutant emission by 29 countries. Source: Authors’ amplification.

**Fig 4:**
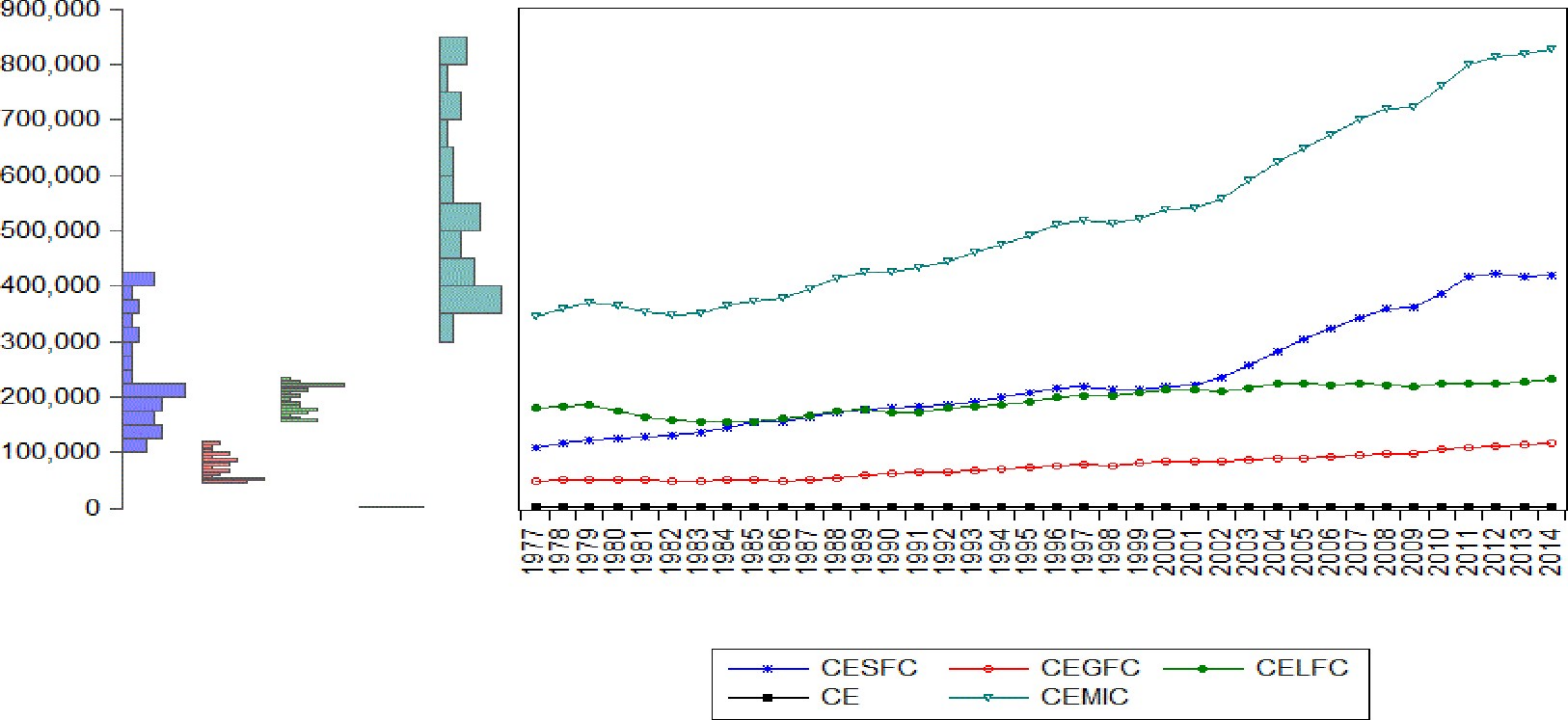
Mean value of pollutant emissions by years. Source: Authors’ amplification.

Table 5 shows the descriptive statistics of the particular variables of high mean value over the period of 1977–2014, countries by the type of pollutant emissions, China (CESFC, CE), USA (CEGFC, CELFC, AET, CSE) and India (AIT) show the highest mean value Fig 3 Although, Morocco (CR), Mexico (MI, ME), Philippine and Canada (ME), Mexico and Panama (MIE), Costa Rica and Argentina (IF) register the lowest mean value. Table 6 indicate the term of matrix correlation, relationships between energy consumption and selected instrumental variables, emissions such as CESFC, CEGFC, CELFC, CE, and CEMIC were noticed. Fig 4 explored the value of the mean, the manufacturing industries, and construction increase continuously comparatively solid, liquid and gaseous fuel consumption. The result computed by GMM method and in order to remove inconvenience, consider stationary test according to cross-section independence in first generation Table 7 unit root test in common root and individual intercept in level and 1^st^ generation and Table 8 with first deference.

**Table 5:** Descriptive statistics (raw data).

Variables	Mean	Median	Max	Min	Sta.Dev.	Skewness	Kurtosis	Jarque-Bera	Prob	Obs
**GDP**	1,080,000 m	309,000, m	16,200,000, m	6,750, m	2,250,000m	4.11	22.03	19,728.63	0.00	1,102
**GDPC**	2.209713	2.26	13.64	-15.32	3.73	-0.73	6.46	646.47	0.00	1,102
**CESFC**	231,943.80	21,536.29	7,499,587.00	-113.68	752,749.80	5.98	47.32	96,758.81	0.00	1,102
**CEGFC**	74,688.62	17,552.10	1,432,767.00	0.00	202,645.60	4.84	26.34	29,322.15	0.00	1,102
**CELFC**	195,177.90	56,612.98	2,494,601.00	1,452.13	411,692.50	4.04	19.65	15,727.17	0.00	1,102
**CE**	525,318.10	114,734.90	10,291,927.00	2,002.18	1,276,136.00	4.23	22.95	21,557.75	0.00	1,102
**CEMIC**	20.54	19.53	49.15	0.00	7.29	0.69	3.77	115.22	0.00	1,102
**ME**	2.15	1.08	28.83	0.00	3.55	4.25	24.91	24,815.98	0.00	1,079
**AET**	943 m	76 m	15,700 m	0.00	2,610 m	3.79	17.03	6,592.15	0.00	622
**MI**	2.13	0.96	27.10	0.00	3.23	3.31	17.06	10,832.16	0.00	1,077
**AIT**	444 m	200 m	5,320, m	0.00	638 m	2.88	13.85	6,421.31	0.00	1,022
**CSE**	34,300 m	10,100 m	721,000 m	13.5 m	70,000 m	5.13	38.34	55,969.20	0.00	992
**IGD**	25.76	4.61	3,057.63	-27.05	176.35	13.00	186.77	1,581,752.00	0.00	1,102
**CR**	0.22	0.00	8.71	0.00	0.66	6.18	58.31	147,507.50	0.00	1,102
**IF**	3.56	2.30	22.08	-2.28	3.66	1.36	4.88	439.85	0.00	964
**MIE**	2.39	2.12	10.67	0.00	1.47	1.04	4.67	316.74	0.00	1,071
**AL**	40.62	44.82	71.54	2.46	18.69	-0.43	2.17	64.52	0.00	1,079

Note: m indicates million. Sources: Definition of variable available in Table 2.

**Table 6:** Matrix correlation.

Prob	GDP	GDPC	CESFC	CEGFC	CELFC	CE	CEMIC	ME	AET	MI	CR	CSE	IGD	CR	IF	MIE	AL
**GDP**	1.00																
**GDPC**	0.022***	1.00															
**CESFC**	0.571***	0.403***	1.00														
**CEGFC**	0.945***	-0.043*	0.433***	1.00													
**CELFC**	0.941***	0.093***	0.606***	0.956***	1.00												
**CE**	-0.280***	0.367***	0.220***	-0.361***	-0.203***	1.00											
**CEMIC**	0.814***	0.294***	0.929***	0.731***	0.855***	0.034*	1.00										
**ME**	-0.061***	-0.092***	-0.034*	-0.097**	-0.118***	-0.299***	-0.074**	1.00									
**AET**	0.799***	-0.021*	0.374***	0.893***	0.897***	-0.274***	0.657***	-0.126***	1.00								
**MI**	-0.096***	-0.094***	-0.028*	-0.134***	-0.153***	-0.215***	-0.085***	0.086***	-0.147***	1.00							
**AIT**	0.121***	0.380***	0.413***	0.064***	0.183***	0.215***	0.343***	-0.002*	0.016*	-0.100***	1.00						
**CSE**	0.837***	-0.056*	0.414***	0.733***	0.659***	-0.378***	0.591***	0.040*	0.520***	0.082**	0.092***	1.00					
**IGD**	-0.040*	-0.113***	-0.052*	-0.061*	-0.046*	0.087***	-0.057*	-0.059*	-0.052*	-0.091***	-0.06*	-0.088***	1.00				
**CR**	0.155***	0.298***	0.553***	0.078**	0.201***	0.221***	0.446***	0.236***	0.034*	-0.096***	0.451***	0.111***	-0.052*	1.00			
**IF**	0.443***	-0.170***	0.049*	0.386***	0.320***	-0.250***	0.188***	0.092***	0.226***	-0.036*	-0.133***	0.503***	-0.021**	-0.029*	1.00		
**MIE**	0.350***	0.118***	0.146***	0.414***	0.444***	0.069*	0.292***	-0.216***	0.518***	-0.283***	0.265***	0.144***	-0.037***	0.015*	-0.093***	1.00	
**AL**	0.137***	0.061*	0.211***	0.088***	0.143***	-0.099***	0.198***	0.275***	0.151***	-0.051*	0.215***	0.162***	-0.04*	0.2***	-0.005*	0.205***	1.00

Sources: Computation by authors. Note: Please see, Table 2 for the variable’s definition

*** specifies the statistically significant at 1% levels.

** specifies the statistically significant at 5% levels.

* specifies the statistically significant at 10% levels

**Table 7:** Unit root of individual variables (level).

Level											
Individual intercept						Individual intercept and trend					
Variables	CR	Individual root			Hadri	CR		Individual root			Hadri
	LLC	IPS	ADF	PP		LLC	Breitung	IPS	ADF	PP	
**GDP**	8.739	13.65	16.039	17.32	19.797***	3.537**	6.503	5.835	31.959**	40.602**	15.174***
**GDPC**	-13.66***	-12.975***	280.248***	380.343***	4.285***	-13.69***	-14.14***	-12.784***	263.21***	425.104***	2.308***
**CESFC**	3.314**	3.172**	49.643**	51.126**	17.197***	1.202**	6.941	2.330**	60.639**	64.380**	13.552***
**CEGFC**	3.025**	6.834	18.342	25.46**	16.917***	4.958	7.018	5.049	41.332**	44.202**	7.476***
**CELFC**	2.695**	3.300**	59.771**	55.412**	16.591***	-1.041**	2.297**	1.086**	59.143**	36.310**	9.302***
**CE**	-4.601***	-1.436**	74.517**	89.044***	16.995***	-0.633**	-0.7237***	-0.783**	67.931**	83.414***	11.111***
**CEMIC**	3.992***	6.072	26.559**	28.959**	17.839***	2.607***	6.2	3.839**	36.388**	39.174**	14.375***
**ME**	1.475**	2.156**	42.353**	68.413**	18.215***	-1.518**	-1.482**	-2.352**	81.068**	102.354***	7.125***
**AET**	-2.149***	-3.04***	79.523	110.819	11.871***	.717**	-5.135***	-1.167***	61.545	87.606	3.010***
**MI**	3.707**	5.879	37.712**	52.798**	18.826***	-2.416**	2.854**	-2.118**	88.14**	113.464***	13.400***
**AIT**	-6.969***	-8.674***	185.301***	248.052***	6.569***	-6.696***	-5.215***	-6.628***	139.403***	203.930***	7.207***
**CSE**	11.033	14.16	3.186	1.533	19.175	2.628**	6.902	5.625	19.432	15.76	14.747***
**IGD**	-5.321***	-6.227***	147.989***	202.607***	2.050**	-6.39***	-5.44***	-6.066***	144.463***	204.946***	7.540***
**CR**	-3.471***	-3.471***	72.654***	117.598***	4.397***	-3.677***	-3.956***	-2.407	61.733**	81.987***	9.117***
**IF**	-2.095***	-2.917***	96.901***	113.746***	11.374***	-6.89***	-3.299***	-4.257***	107.359***	127.867***	127.867***
**MIE**	-3.048**	-0.505**	62.802**	60.849**	15.849***	-1.574***	-1.968***	-0.695	60.823	68.363	8.344***
**AL**	-1.654**	3.599**	39.238**	53.892**	14.337***	-0.876**	1.459**	1.960**	38.680**	45.758**	12.985**

Source: Computation by authors. Note: Please see, Table 2 for the variable’s definition

*** specifies the statistically significant at 1% levels.

** specifies the statistically significant at 5% levels.

* specifies the statistically significant at 10% levels.

**Table 8:** Unit root of individual variables (first difference).

First difference											
Individual intercept						Individual intercept and trend					
Variables	CR	Individual root			Hadri	CR		Individual root			Hadri
	LLC	IPS	ADF	PP		LLC	Breitung	IPS	ADF	PP	
**GDP**	-6.892***	-9.509***	217.311***	321.655***	14.3024***	-11.7028***	-7.733***	-11.631***	229.986***	379.76***	9.978***
**GDPC**	-26.19***	-29.252***	692.647***	771.738***	-5.267**	-23.208***	-15.864***	-26.604***	686.887***	5352.97***	9.362***
**CESFC**	-12.81***	-18.857***	430.863***	650.227***	7.574***	-11.713***	-6.668***	-18.948***	431.265***	970.338***	2.286**
**CEGFC**	-8.981***	-11.885***	293.098***	593.465***	4.065***	-10.029***	-2.635***	-11.885***	243.819***	1059.95***	3.771***
**CELFC**	-11.43***	-14.09***	311.425***	584.042***	2.304***	-10.102**	-7.062***	-12.884***	269.772***	1016.83***	7.214***
**CE**	-15.56***	-20.566***	474.701***	779.588***	.8016**	-13.235**	-13.79***	-19.213***	429.375***	1259.51***	4.450***
**CEMIC**	-9.513***	-14.738***	334.107***	65.894***	10.289***	-8.196***	-5.221***	-13.383***	281.423***	688.814***	3.978***
**ME**	-14.77**	-20.001**	462.271***	793.076***	-0.17**	-12.353***	-10.094**	-18.622***	389.420***	1483.56***	4.355***
**AET**	-6.224***	-11.817***	226.406***	488.133***	5.059***	-1.429**	-4.816***	-7.762***	175.094***	906.84***	25.403***
**MI**	-11.5***	-20.324***	468.653***	745.406***	2.193**	-7.822***	-4.919***	-18.521***	410.287***	2596.82***	5.858***
**AIT**	-19.22***	-22.654***	520.978***	782.556***	-1.269***	-15.856***	-8.849***	-17.968***	426.592***	3790.04***	4.138***
**CSE**	-10.58***	-14.029***	318.835***	551.193***	12.867***	-11.013***	-8.543***	-13.078***	330.713***	863.553***	5.204***
**IGD**	-22***	-24.622***	589.251***	790.052***	3.526***	-19.072***	-14.156***	-22.111***	486.624***	3147.26***	23.308***
**CR**	-21.31***	-25.57***	552.546***	653.620***	-2.768***	-18.106***	-15.786***	-23.328***	468.263***	656.767***	1.455**
**IF**	-22.4***	-19.626***	442.645***	740.570***	.0.187**	-29.65***	-11.275***	-16.375***	361.590***	1509.40***	6.791***
**MIE**	-12.71***	-14.969***	326.141***	635.900***	3.653***	-10.897***	-11.559***	-12.816***	262.854***	1462.23***	16.187***
**AL**	-8.498***	-12.687***	291.138***	559.093***	5.3833***	-7.563***	-5.376***	-10.829***	238.115***	796.856***	7.260***

Source: Computation by authors. Note: Please see, Table 2 for the variable’s definition

*** specifies the statistically significant at 1% levels.

** specifies the statistically significant at 5% levels.

* specifies the statistically significant at 10% levels.

As we notice the variables are non-stationary in their level and become stationary after 1^st^ difference. [56–58]

### 4.2 Panel regression analysis

Panel regression indicate the GMM a regression method with AB in n-step. In the GMM estimation, the explanatory variable individually estimated regression with dependent variables. The panel data study by providing the solution of common problems in different developed and developing countries; the heterogeneity of behavior of the individual explanatory variable, the endogenous and simultaneity by bidirectional causality problem. This research paper will estimate a dynamic model (where the endogenous variables are included as explanatory variables along with more than one lag). The white period method applies for the coefficient covariance method individually for computation of CESFC, CEGFC, CELFC, CE, and CEMIC with other explanatory variables. The difference cross-sectional period was used for cross section in none period, the GMM iterations was computing in 2-step, that varies by cross-section in the white period.

According to Sargan statistic, all estimated models are statistically highly significant, and the value of J-Statistic, that could be explained between 5.08 and 17.31 of the variability in pollutant emission. Hence in the model, where the same number in instrument as a parameter, the optimized value of the objective function is zero. If the number of the instruments increased than parameters, the optimized value will be greater than zero, and the J-statistic used as the test of over-identifying moment condition. The J-statistics and instrumental rank, reported by Sargan statistics, where the instrumental rank greater in the individual model, than the number of estimated coefficients, we may use to construct Sargan test over the identifying restrictions. While in the null hypothesis over-identifying restriction is valid, the J-statistic in panel equation is different from the ordinary equation, where the Sargan statistics are distributed as a *χ*(*ρ*−*k*). Where the estimated coefficient is k and instrumental rank is *ρ* individual in each model. The Sargan test was computed in CESFC by scalar *pval* = @chisq (8.50,9.0) individually. The related coefficient of GDP per capita and squared GDP per capita are statistically significant in all estimated model, except model 4, the EKC hypothesis is confirmed in case of CE negatively impact. Furthermore, estimated regression appears to fit the data by the value of the Sargan test, they can explain all most 10% to 82% of the pollutant emission. The inverted U-Shaped curve emerges in all cases of harming secretions, except CE, with regard of GDPSQ, MI, AIT, CSE, IGD, IF, ME and AL; knowledge that expectation ecological damage reduction is not supported positively in estimated models, show a negative influence on pollutant emission. Also, we notice with some exceptional the renewable energies consumption reduces the pollution emission, like the higher GDP implies higher production and more insurance and financial services acquired [59]. In the term of merchandise export (ME) like [60]. The results of the variables employed to control for the scale effect and pollution conditions.

**Fig 5:**
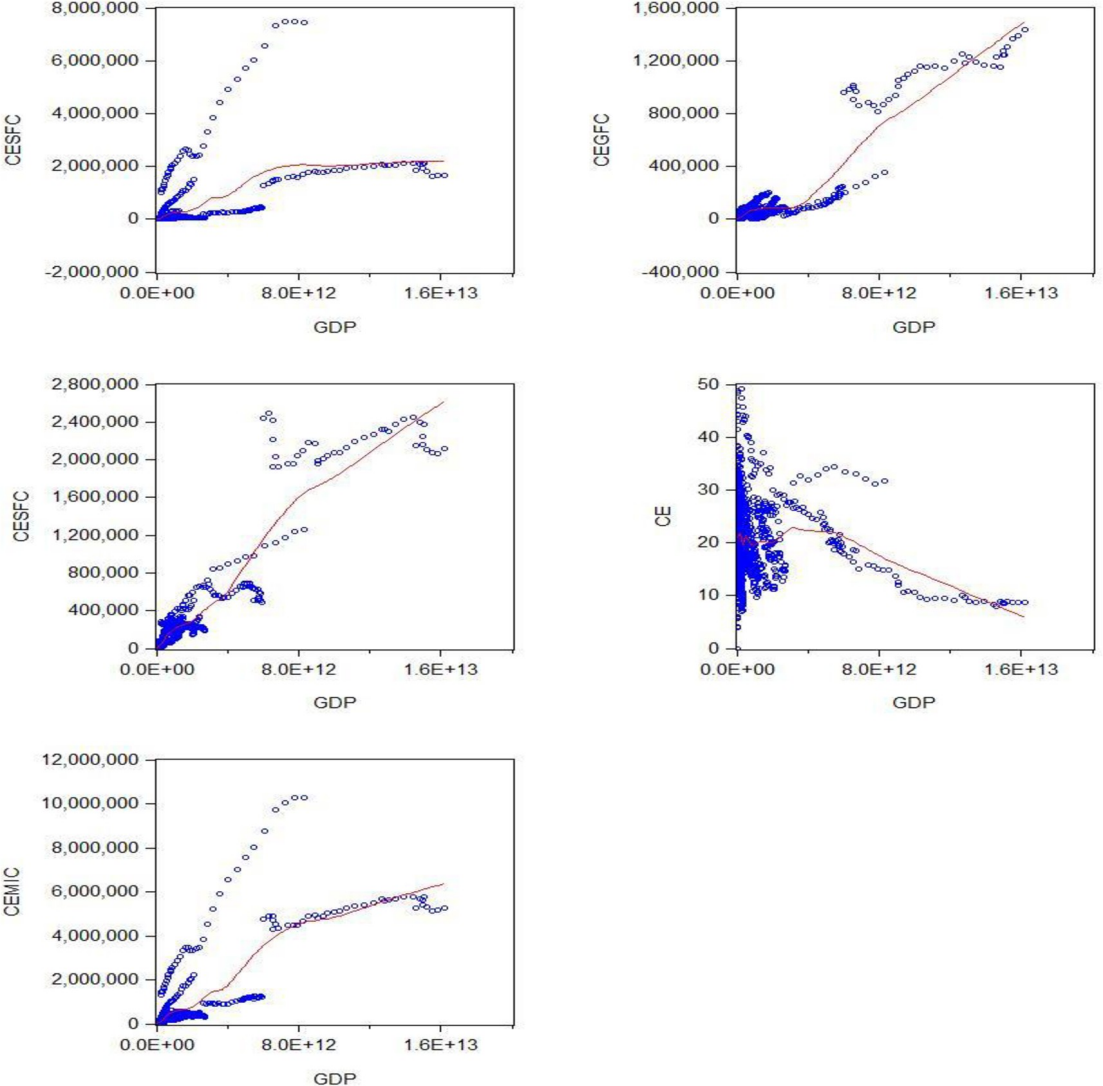
Plotted graph between GDP per capita and CESFC, CEGFC, CESFC, CE and CEMIC. Source: Authors’ amplification.

Fig 5 reveals the plotted graphs between GDP and pollutant emission. The EKC hypothesis appaired to be sustained since the inverted U-shaped curve tends to be fit properly in CESFC, and also indicated the sequence of U-shaped, in the term of CEGFC and CESFC, curve straightly going upward and we notice that the turning points are not in line. Hence in carbon emission the EKC curve coming down and notice that after high technology in industries and export reduce the level of EKC. In the last CEMIC the intensity of emission continuously in developing countries. Furthermore, [61] specified a higher likelihood of identifying turning points in the case of developed to developing countries.

### 4.3 Co-integration and causal investigation

In the co-integration, the Padroni panel test is explored in Table 9. The dimensional approach of statistics, the autoregressive coefficient in the different developed and developing countries [53,62] for the unit root test on the estimated residual consideration for heterogeneity across the country and time factor. And the analysis of long-run cointegration relationships has been taken from developed and developing countries in the modern series analysis.

**Table 9:** Pedroni (Engle-Granger based) test.

Panel A: Wintin-dimension						
Panel co-integration test	Individual intercept		Individual intercept and trend		No intercept or trend	
	Statistic	Weighted Statistic	Statistic	Weighted Statistic	Statistic	Weighted Statistic
Panel v-Statistic	2.737***	-3.115*	22.167***	-0.938*	-1.480*	-3.404*
Panel rho-Statistic	0.658*	1.524*	-1.226*	2.162*	-0.248*	1.393*
Panel PP-Statistic	-0.388*	2.749*	-3.865***	0.195*	-0.144*	1.822*
Panel ADF-Statistic	-0.242*	2.993*	-3.652***	4.061	-0.235*	1.105*
Panel B: Between- dimension						
Panel co-integration test	Individual intercept		Individual intercept and trend		No intercept or trend	
	Statistic		Statistic		Statistic	
Group rho-Statistic	3.275*		4.086		2.660*	
Group PP-Statistic	1.776*		0.617*		1.635*	
Group ADF-Statistic	2.981*		0.236*		2.977*	

Source: Computation by authors. The lag length was selected by Schwarz Info criterion.

Note: Please see, Table 2 for the variable’s definition

*** specifies the statistically significant at 1% levels.

** specifies the statistically significant at 5% levels.

* specifies the statistically significant at 10% levels.

The Padroni panel test in panel A, ADF statistically reject the null hypothesis of no co-integration with individual intercept, trend and No intercept or trend. The statistically mean value of individual autoregressive coefficient related with unit root test of individual each developed and developing the state. In the panel B, the co-integration employed with rho, PP and ADF statistics, and explored by the Kao Table 10 in Engle-Granger based test, the ADF (t-statistics) is 2.490 (sig) with residual variance. Where the vector of co-integration is homogenous in different states. The result provides the hypothesis of co-integration of developing and developed states variables.

**Table 10:** Kao (Engle Granger based) test.

ADF (t-Statistic)	Residual variance	HAC variance
2.490***	8.24E+21	2.64E+22

Source: Computation by authors. The lag length was selected by Schwarz Info criterion.

*** specifies the statistically significant at 1% levels.

** specifies the statistically significant at 5% levels.

* specifies the statistically significant at 10% levels.

The third test is a Fisher, that approach is used to underlying Johansen methodology by panel co-integration test [63], showed in Table 11. This panel co-integration test aggregates with the p-value of individual Johansen trace statistics and eigenvalue [64]; also reject the null hypothesis of no cointegration.

**Table 11:** Fisher (Combined Johansen) test.

Hypothesized No. of CE(s)	Fisher Stat.* (from trace test)	Fisher Stat.* (from max-eigen test)
None	135.8***	102.0***
At most 1	64.86***	61.32***
At most 2	32.51*	32.51*

Source: Computation by authors. The lag length was selected by Schwarz Info criterion and Probabilities are computed using asymptotic Chi-square distribution

*** specifies the statistically significant at 1% levels.

** specifies the statistically significant at 5% levels.

* specifies the statistically significant at 10% levels.

Onward, since the variables are co-integrated, a panel vector error correction model is estimated in order to perform Pairwise Granger Causality test Table 12, we reject the null that GDPC does not Granger cause CEMIC, and also in the opposite direction.

**Table 12:** Pairwise Granger causality tests.

Null Hypothesis:	Obs	F-Statistic
CEMIC does not Granger Cause GDPC	1073	13.732***
GDPC does not Granger Cause CEMIC		47.520***
AIT does not Granger Cause GDPC	965	16.161***
GDPC does not Granger Cause AIT		4.293***
CSE does not Granger Cause GDPC	961	1.510
GDPC does not Granger Cause CSE		11.346***
CR does not Granger Cause GDPC	1073	21.069***
GDPC does not Granger Cause CR		5.530***
AIT does not Granger Cause CEMIC	965	56.007***
CEMIC does not Granger Cause AIT		6.348***
CSE does not Granger Cause CEMIC	961	133.750***
CEMIC does not Granger Cause CSE		22.872***
CR does not Granger Cause CEMIC	1073	51.272***
CEMIC does not Granger Cause CR		3.889***
CSE does not Granger Cause AIT	863	0.498
AIT does not Granger Cause CSE		3.675***
CR does not Granger Cause AIT	965	4.190***
AIT does not Granger Cause CR		3.319**
CR does not Granger Cause CSE	961	0.009
CSE does not Granger Cause CR		0.929

Source: Computation by authors. The lag length was selected by Schwarz Info criterion.

Note: Please see, Table 2 for the variable’s definition

*** specifies the statistically significant at 1% levels.

** specifies the statistically significant at 5% levels.

* specifies the statistically significant at 10% levels.

**Fig 6:**
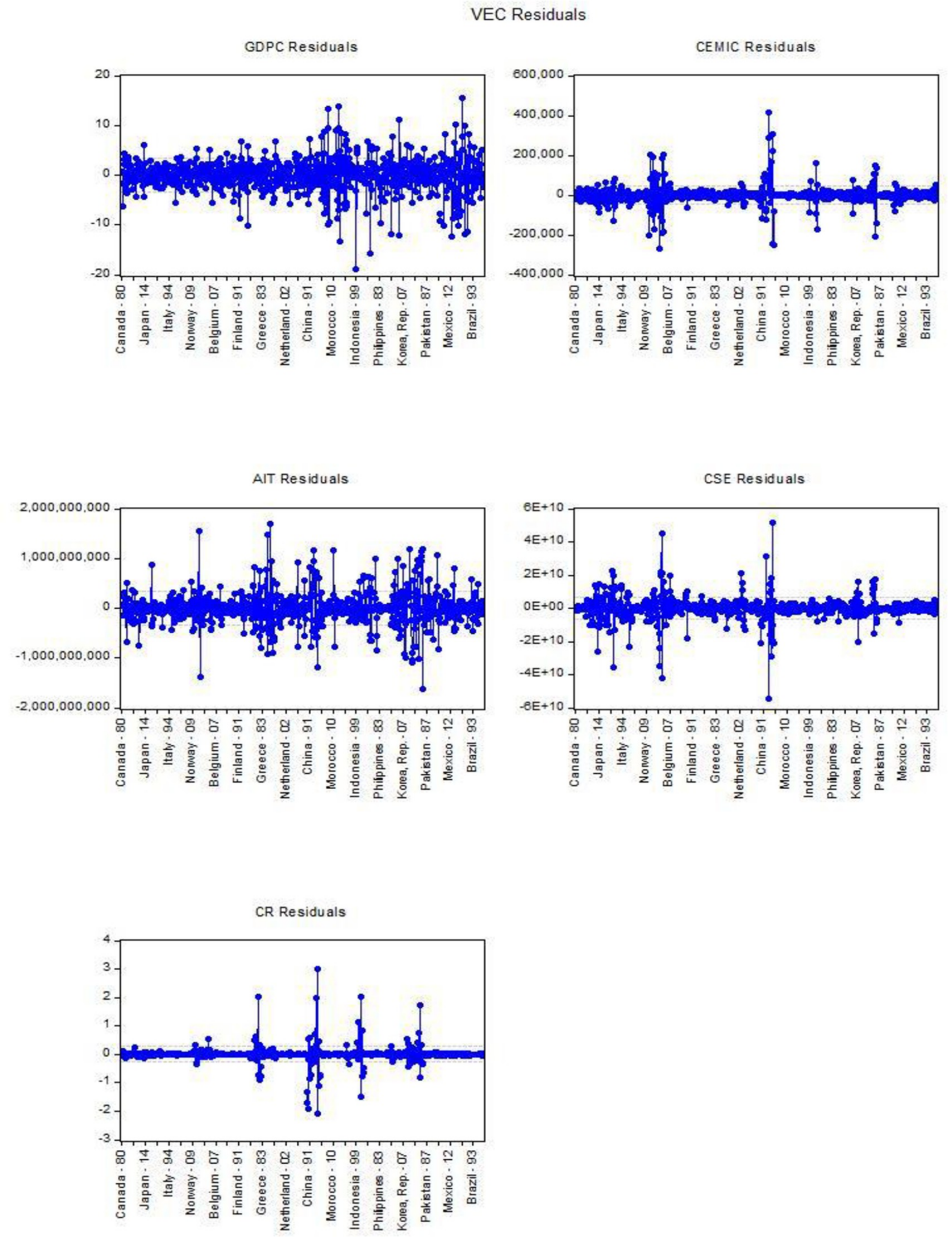
VEC residuals by states. Source: Authors’ amplification.

Table 13. Indicate Vector Error Correction (VEC), with cointegration restrictions B (1,1) = 1 and the convergence attained after 1 iteration with t-statistics and Standard error Fig 6. The specification of VEC has five (*k* = 5) endogenous variables, GDPC, CEMIC, AIT, CSE and CR, the exogenous intercept C(d = 1) and lags include 1 to 2 (*p* = 1). Thus, there is (kp+d = 6) regression of each of the three-equation in the VEC individually.

**Table 13:** Vector error correction model.

Error Correction:	Cointegration	Standard error	t-statistics	R-squared	F-statistic
D(GDPC)	-0.033	-0.01641	-2.04368	0.162128	26.05806
D(CEMIC)	2034.459	-209.35	9.71799	0.690684	300.7023
D(AIT)	1093653	-1525124	0.71709	0.049723	7.046426
D(CSE)	4.62E+08	-3.20E+07	14.2740	0.291235	55.33518
D(CR)	-0.002747	-0.00132	-2.08210	0.069086	9.994087

Source: Computation by authors. The lag length was selected by Schwarz Info criterion in cointegration restriction. Note: Please see, Table 2 for the variable’s definition

**Fig 7:**
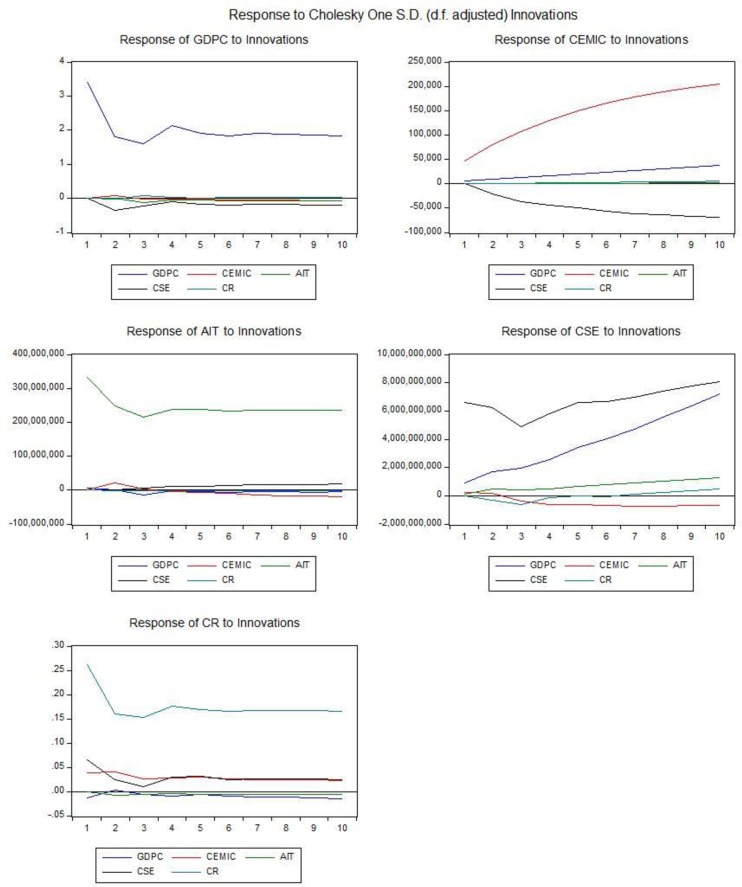
Impulse response. Source: Authors’ amplification.

The effect of CEMIC has also been investigated by using impulse response by Cholesky one S. D (d.f. adjusted) innovation in decomposition method Fig 7, the impulse response of emission shock to Eqs 3–4 individually. The level of significance impulse function has been investigated at 95%. The result from variance decomposition indicate the individual variables effects. In order to measure the deviation method, which impulses to GDPC are explained by CEMIC, AIT, CSE, and CR. Eq 4 according to VAR lag order selection criteria the endogenous variables indicated significant relationship in lag-2 at Schwarz information criteria (SC) and lag-17 at Hannan-Quinn information criteria, the CO2 emission is not too much efficient in lag-17, therefore the Johansen Fisher Panel Cointegration Test is applied in lag (2–1 = 1), it indicates the significant p-value (0.000) in model Table 13 are cointegrated in that case we use Vector Error Correction Estimates (VECM) in lag-1 with cointegration restrictions. The t-test in error correction model indicate significant relationship among GDP per capita and manufacturing industries and construction (CEMIC) with 9.718 which is more than 1.96, concerning Eq 4 identify that 69.0% manufacturing industries and construction have the influence on the level of GDP per capita with F-statistics (300.702) comparatively others. In Eq 5 noticed the statistically insignificant influence on arms import (AIT) with 4.9% by GDP per capita. Hence, the commercial service export (CSE) also indicate the significant relationship with GDP per capita in Eq 6, 29.123% has the influence on the level of GDP per capita with F-statistics (55.335). Eq 7 indicates the coal rent (CR) has not too influence on GDP per capita with 6.90%. Moreover, the vector error correction term statistically significant in two endogenous variables, the analysis suggests that the above explanatory variables Table 13. are the main sources of volatility in different states by GDP per capita.

## 5. Conclusion

The objective of this research study was to determine the EKC hypothesis and afterward the causal relationships between carbon emission solid, liquid and gases fuel, merchandise export, economic growth, arms export trend, coal rents, and military expenditure, for a panel consisting of 29 countries the period 1977–2014.

In the panel data, we noticed cross-sectional dependence in each of the variables, we employed the Generalized Method of Movement/Dynamic Panel data, the transformation of first deference with white period instrumental weighted mix. The results of GMM regression confirmed the acquired hypothesis for emission of CO2 emission from liquid fuel consumption, CO2 emission from manufacturing industries, where the outcome of GMM estimation corroborated, furthermore the EKC approach for solid, liquid and fuel consumption emission and CO2 emission.

Moreover, the estimation of GDP per capita with a panel vector error correction model in order to performed Pairwise Granger Causality test. The model shows a short run unidirectional causality from GDP per capita growth to CO2 emission from manufacturing industries and construction, arms import, commercial service export, and coal rents, as well as a causal link between manufacturing industries, arms import, commercial service export and coal rent.

Likewise, the neoclassical view was endorsed in developing and developed countries, respectively the hypothesis impartiality. The main implication instigating from this research can be followed: 29 developed and developing countries should promote the use of renewable vitalities that are constantly restocked and which will not directly be diminished. Hence, the use of renewable vitalities will contribute to the decrease in GHGs emission.

Besides, 29 developed and developing countries may benefit from enhanced social stability, job opportunity by modernized technologies. Finally, as endeavors of future research, our aim to outspread the empirical analysis in order to verify and test the EKC hypothesis employing the environmental performance and encourage to developed countries to secure the environment especially for arms and huge manufacturing industries.
